# Soil moisture evaluation over the Argentine Pampas using models, satellite estimations and in-situ measurements

**DOI:** 10.1016/j.ejrh.2020.100723

**Published:** 2020-10

**Authors:** P.C. Spennemann, M.E. Fernández-Long, N.N. Gattinoni, C. Cammalleri, G. Naumann

**Affiliations:** a Consejo Nacional de Investigaciones Ciencia y Tecnología (CONICET)-Servicio Meteorológico Nacional (SMN), Buenos Aires, Argentina; b Universidad Nacional de Tres de Febrero (UNTREF), Buenos Aires, Argentina; c Facultad de Agronomía de la Universidad de Buenos Aires (FAUBA), Argentina; d Instituto Nacional de Tecnología Agropecuaria (INTA), Instituto de Clima y Agua, Argentina; e European Commission, Joint Research Centre, Ispra, Italy

**Keywords:** Soil moisture, Evaluation, Land Surface Models, Satellite estimations

## Abstract

**Study region:**

The Pampas region is located in the central-east part of Argentina, and is one of the most productive agricultural regions of the world under rainfed conditions.

**Study focus:**

This study aims at examining how different Land Surface Models (LSMs) and satellite estimations reproduce daily surface and root zone soil moisture variability over 8 in-situ observation sites. The ability of the LSMs to detect dry and wet events is also evaluated.

**New hydrological insights for the region:**

The surface and root zone soil moisture of the LSMs and the surface soil moisture of the ESA CCI (European Space Agency Climate Change Initiative, hereafter ESA-SM) show in general a good performance against the in-situ measurements. In particular, the BHOA (Balance Hidrológico Operativo para el Agro) shows the best representation of the soil moisture dynamic range and variability, and the GLDAS (Global Land Data Assimilation System)-Noah, ERA-Interim TESSEL (Tiled ECMWF’s Scheme for Surface Exchanges over Land) and Global Drought Observatory (GDO)-LISFLOOD are able to adequately represent the soil moisture anomalies over the Pampas region. In addition to the LSM results, also the ESA-SM satellite estimated anomalies proved to be valuable. However, the LSMs and the ESA-SM have difficulties in reproducing the soil moisture frequency distributions. Based on this study, it is clear that accurate forcing data and soil parameters are critical to substantially improve the ability of LSMs to detect dry and wet events.

## Highlights

• The Pampas region, located in central Argentina, is one of the most productive rainfed agricultural regions of the world.
• The ability of the Land Surface Models (LSMs) and satellite estimations in reproducing daily surface and root zone soil moisture is evaluated.
• BHOA is able to correctly represent the soil moisture dynamic range and variability, and the GLDAS-Noah, ERA-Interim and LISFLOOD are able to adequately represent the soil moisture anomalies over the Pampas region.
• In addition to the LSM results, also the ESA CCI satellite estimations anomalies proved to be valuable.
• Accurate forcing data and soil parameters are critical to improve substantially the ability of LSMs to detect dry and wet events.

## 1 Introduction

Agricultural production is one of the economic sectors most vulnerable to climate variability and long-term changes (Verón et al., 2015; Meinke et al., 2006). Argentina is one of the world's major agricultural exporters, ranking among the top oilseed and cereal grains producers (Calviño and Monzón, 2009). Crops in central eastern Argentina (i.e. Pampas region) are mostly cultivated under rainfed conditions. Thus, the main climatic constraint on agricultural production is the water supply (Hall et al., 1992; Maddonni, 2012; Aramburu Merlos et al., 2015). As an example, during the recent 2017−18 drought the country exports of maize and soybean were reduced by more than US$ 4.8 billion (Bolsa de Cereales, 2018a, 2018b). Such significant losses affect not only the local economy but also global commodity markets and food prices (FAO, GIEWS-2018), which could lead to food insecurity in most vulnerable countries. This alone justifies the global and regional efforts carried out by different institutions and organizations (e.g. the WMO Regional Climate Center for southern South America - RCC-SSA, or the European Commission’s Global Drought Observatory - GDO) to improve the monitoring and forecasting of the key variables that define droughts.

Currently, the main regional and global drought monitoring centers covering Argentina (e.g. Climate Prediction Center, GDO) use drought indices based on precipitation, soil moisture estimates from Land Surface Models (LSMs) and/or satellite and remotely-sensed vegetation statuses, as individual indicators or as combined drought indicators (Sepulcre-Canto et al., 2012). Precipitation is a key indicator for monitoring water conditions in the soil, as it is the main driver of deficit or excess conditions. The propagation of these anomalies through the terrestrial segment of the hydrological cycle, as well as the impacts on vegetated land, is commonly analysed using soil moisture and vegetation indicators. During the monitoring phase, accurate estimations of soil moisture anomalies can inform rain-reliant farmers about potential production impacts during the different crop growth stages (i.e. sowing, flowering).

It is clear that soil moisture is a key variable for monitoring the evolution of excess or deficit water conditions. As such, it is important to describe the strengths and limitations of the main available soil moisture data sources: 1) Satellite microwave estimations, which have an extensive spatial coverage but they can only estimate surface (i.e., first few centimetres) soil moisture; 2) LSMs outputs, which have good temporal and spatial coverage, but strongly depend on the quality and availability of forcing variables (e.g., precipitation, shortwave radiation) as well as on soil and vegetation parameters; 3) In-situ measurements, which are the most accurate values of soil moisture for a given location, however sparseness of the acquisitions and spatial heterogeneity hinder the representativeness of local in-situ measurements for large areas. Thus, in order to perform a consistent lite microwave estimations, which have an extensive spatial coverage but they can only estimate surface (i.e., first few centimetres) soil moisture; 2) LSMs outputs, which have good temporal and spatial coverage, but strongly depend on the quality and availability of forcing variables (e.g., precipitation, shortwave radiation) as well as on soil and vegetation parameters; 3) In-situ measurements, which are the most accurate values of soil moisture for a given location, however sparseness of the acquisitions and spatial heterogeneity hinder the representativeness of local in-situ measurements for large areas. Thus, in order to perform a consistent evaluation of soil moisture from LSMs and/or satellite estimations, several observational sites with one or several in-situ measurements (i.e. networks) are needed.

Previous studies using soil moisture networks, assessed the capability of LSMs and satellite soil moisture estimations over different regions (e.g. Europe, North America, Africa and China), and found that, in general, LSMs and satellite estimations are able to reliably reproduce the seasonal cycle (Albergel et al., 2012; Zhang et al., 2018). In particular, Albergel et al. (2012) evaluated the ECMWF TESSEL (European Centre for Medium-Range Weather Forecasts, Tiled ECMWF’s Scheme for Surface Exchanges over Land) LSM model, forced by ERA-Interim data (Dee et al., 2011), against soil moisture networks from several countries and regions (e.g. Spain, France, Australia and West Africa). The authors also concluded that ERA-Interim (i.e. TESSEL LSM) surface soil moisture is well represented in those regions. However, they highlight that, over some regions the modelled surface soil moisture has a lower variability (or smaller dynamical range) than the in-situ measurements. Also, a lower skill is observed over dry lands (e.g. AMMA network in western Africa). The authors observed that the root zone soil moisture (i.e., top 1 m of soil) analysed over 48 stations, shows good correlation with in-situ measurements.

In a similar research line, several global studies focused on determining the performance of satellite soil moisture estimations such as the ESA CCI (European Space Agency Climate Change Initiative, hereafter referred to as ESA-SM) daily surface combined soil moisture product (Dorigo et al., 2017), which is a combination of the different active and passive microwave sensors from 1978-onwards. The study of Dorigo et al. (2012) analysed global trends of surface soil moisture based on ESA-SM, ERA-Interim and the Noah LSM from the Global Land Data Assimilation System (GLDAS, Rodell et al., 2004), on monthly and seasonal scales. Highlighting the consistency across datasets, they also pointed out a good agreement with the sign of precipitation and vegetation indices trends. The study of Fang et al. (2016) documented that ESA-SM soil moisture shows a good agreement with 6 in-situ measurements, but over many observational networks the LSM simulations driven by observed precipitation performed better than the ESA-SM (v02.2) soil moisture product. In Cammalleri et al. (2017) a monthly anomaly evaluation of ESA-SM, LISFLOOD LSM (Roo et al., 2000) simulations and satellite land surface temperature as a proxy of soil moisture was performed. They concluded that ESA-SM performed better over dry areas meanwhile LISFLOOD was more reliable over regions with dense meteorological ground stations.

Over Argentina, few studies evaluated LSM simulations and/or satellite estimations of soil moisture (e.g., Fernández Long et al., 2012; Spennemann et al., 2015) with most of them based only on cross comparisons with proxy data (e.g. Standardized Precipitation Index, SPI). For example, the study of Spennemann et al. (2015) evaluated four LSMs from GLDAS against different SPI aggregation periods. The authors concluded that the GLDAS LSMs, in particular the Noah LSM (Ek et al., 2003), are capable of representing wet and dry soil moisture anomalies on monthly time scales (1980−2008). Using the BHOA (“*Balance Hidrológico Operativo para el Agro”*- Operational water balance for agriculture applications, Fernández Long et al., 2012) water balance model performed an evaluation using the Water Requirement Satisfaction Index (WRSI, Senay and Verdin, 2003) against crop yields over the Pampas region, Argentina. The results showed that the model was able to represent the relationship between WRSI and crop yields in a satisfactory way.

Only few studies using in-situ measurements were performed over Argentina (e.g. Ferreira et al., 2011; Grings et al., 2015). In particular, Grings et al. (2015) performed an evaluation of two different satellite estimations (i.e. Advanced Scatterometer (ASCAT) and Soil Moisture Ocean Salinity (SMOS)), the GLDAS-Noah LSM against 16 months of daily in-situ measurements from the Argentinian Space Agency (CONAE) in central Argentina (Córdoba). They also compared the results against SPI to analyse the relationship of the different soil moisture estimations during extreme wet and dry conditions. The GLDAS-Noah showed high correlations against in-situ measurements, followed by SMOS passive estimations. However, SMOS estimations showed a greater dynamic range, while GLDAS-Noah showed a systematic underestimation of the observed soil moisture. Finally, the authors concluded that the three products (i.e. GLDAS-Noah, SMOS and SPI-1 month) are capable of determine extreme deficit and excesses conditions over the region.

The above brief overview highlights the lack of soil moisture evaluations using LSMs and satellite estimations against in-situ observational sites, with records longer than one year, and using several sites over Argentina. This is mainly due to the absence of extensive in-situ measurement networks. This study aims at narrowing this gap by using several observation sites and an observational soil moisture network developed by the CONAE over the Pampas region. Therefore, the main objective of this study is to evaluate the performance of four LSMs and the ESA-SM soil moisture over multiple sites and over an observational network with daily in-situ measurements located within the Pampas region and determining the ability/capability of these soil moisture datasets in detecting dry and wet events. The structure of this study is as follows: Section 2 presents the methods and the different SM datasets; results are presented in Section 3, the discussion in Section 4, and finally the conclusions in Section 5.

## 2 Materials and methods

### 2.1 Methodology

The analysis of the performance of both the LSMs and the satellite datasets are based on the comparison between the in-situ measurements and the estimates on the nearest grid point. The error metrics include the BIAS (simulation – in-situ measurements) indicator, to quantify the systematic errors, the Root Mean Square Error (RMSE) to provide an indication of the mean deviation of the simulated values of soil moisture compared to the observed values, and the unbiased RMSE (unbRMSE) to represent the non-systematic errors (i.e., the RMSE removing the BIAS). Since, soil moisture is usually not normally distributed (Mo, 2008), the non-parametric Spearman correlation was used. The Spearman correlation metric is essentially a Pearson correlation coefficient but computed using the ranks of the data (Wilks, 2011).

The surface soil moisture depth varies among the LSMs (see Table 1), for example the surface layer (root zone) depths range from 0−7 cm (0−45 cm) to 0−15 cm (0−150 cm), and it is variable in the case of satellite estimations. Thus, in order to make the different metrics comparable, the BIAS, RMSE and unbRMSE were divided by the mean soil moisture values of the corresponding LSM/satellite estimation. In this sense, the different metric errors are relative to the different mean values (expressed in %) corresponding to the different soil depths. This also allows a more direct comparison between the LSM metrics (mm units in this study) and the satellite estimations (m^3^/m^3^).

**Table 1:** Main characteristics of the different soil moisture datasets used in this study.

Soil Moisture Dataset	Type	Temporal delay	Location	Soil layer depth (cm)	period	Spatial resolution	Temporal resolution
GLDAS 2.1 Noah	model reanalysis	months	Global	0−10, 10−40, 40−100 and 100−200	2012−2017	0.25°	Daily
LISFLOOD	operational model	near real time	Global	Root zone (variable)	2012−2017	0.1°	Daily
ERA Interim	model reanalysis	months	Global	0−7, 7−21, 21−72, and 72−189	2012−2017	1.0°	Daily
BHOA	operational model	near real time	Argentina	Surface and bottom layers	2012−2017	Local scale	Daily
ESA-SM	satellite	months	Global	Surface (variable)	2012−2016	0.25°	Daily

The soil moisture anomalies (i.e. without the seasonal effect) were constructed using a moving window of 35 days (+/– 17 days), as a departure from the 5 weeks mean values and then scaled by the standard deviation from the same time window (Albergel et al., 2012). This procedure of calculating the soil moisture anomalies using relative short time records is able to reasonable reproduce the percentile distribution frequency of longer records (Ford et al., 2016). In our case, a minimum of 5 records during this 35 days period was required to calculate the anomalies. When this criterion was not fulfilled that specific time was not considered in the analysis.

Taking into account the differences in depths and LSMs, the soil moisture and soil moisture anomalies were standardised to make the different Kernel Density Functions comparable. In that sense, the following standardization was applied

(1) $${SM}_{stand}=\frac{SM-{SM}_{5}}{{SM}_{95}-{SM}_{5}}$$

where SM_stand_ corresponds to standardized soil moisture, SM_5_ and SM_95_ correspond to the 5th and 95th percentiles of soil moisture respectively. Thus, all standardized soil moisture values fall approximately within the 0–1 range.

To quantify the ability of the LSMs to characterize both surplus and deficit soil moisture conditions, all the time series were divided in terciles (upper (67 %–100 %), middle (34 %–66 %) and lower (0 %–33 %)). Then, a Hit for dry events was considered when a LSM simulation showed a value at the lowest tercile that was also observed (i.e. Hit rate). An error was considered when the observation showed a value at the lowest tercile but the LSM showed a value in the upper tercile (i.e. False Alarm). The same criterion was applied for the detection of wet events.

### 2.2 In situ measurements

The CONAE soil moisture observational network consists of 66 sites and 100 sensors distributed in the Pampas region. The sensors used are Hydra Probe II (Stevens®^1^) with hourly time steps at soil depths of 5-cm and/or 50-cm depending on the site. The original soil moisture measurements are in volumetric units (m^3^/m^3^), which are converted to absolute values of column of water in the soil (mm) to make them comparable to the LSMs outputs. The 5-cm and 50-cm measurement depths are representative of 0−10 cm and 0−100 cm soil layers (e.g. Albergel et al., 2012, 2008). The original volumetric units were kept when comparing against the satellite estimations.

**Fig. 1:**
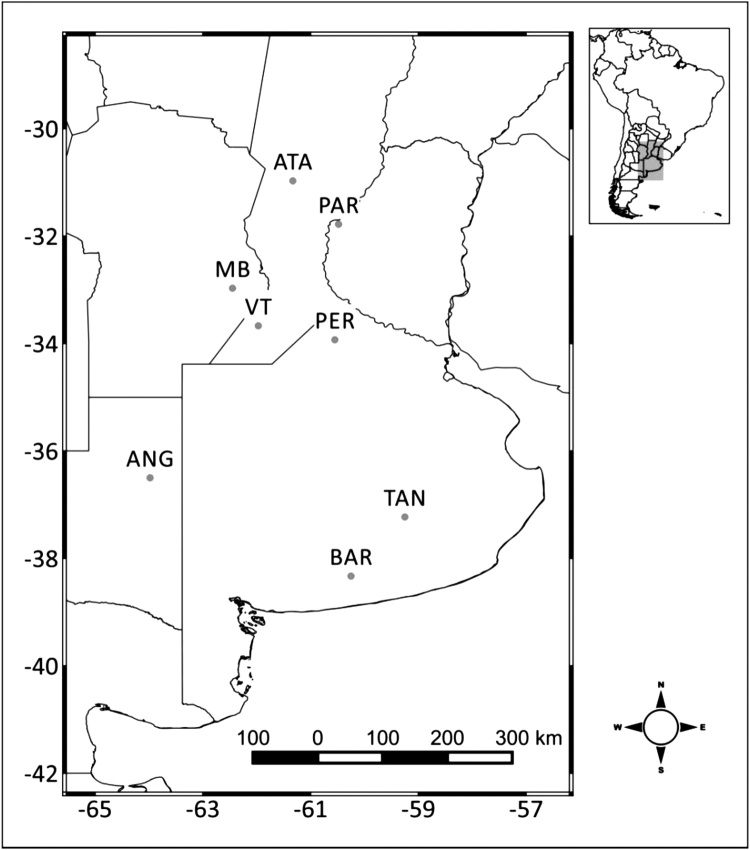
Location of the in-situ measurements: Paraná (PAR), Pergamino (PER), Tandil (TAN), Anguil (ANG), Barrow (BAR), Venado Tuerto (VT), Ataliva (ATA) and Monte Buey (MB).

With the aim to perform the analysis on a dataset of consistent and long ground records, only the sites with at least 1 year of in-situ measurements (not necessarily consecutive days) were used in this analysis. Following this criteria, 8 sites were selected for this study (see spatial location in Fig. 1 and details in Table 2). The hourly in-situ measurements were aggregated to daily data considering only days with less than 33 % of missing hourly data (i.e. maximum of 8 hourly measurements missing). The data were averaged over 12UTC to 12UTC time in order to be comparable with some of the operational LSMs (e.g. BHOA).

**Table 2:** Main characteristics of the in-situ measurement sites used in this study.

Site name	Location	Soil layer depth (cm)	Considered period	Spatial resolution	Temporal resolution
Tandil (TAN)	Lat: 37.23 °S; Long: 59.25 °W	5	06/26/2013-10/22/2014	Local scale	Hourly
Pergamino (PER)	Lat: 33.93 °S; Long: 60.55 °W	5	05/20/2013-10/17/2014	Local scale	Hourly
Barrow (BAR)	Lat: 38.33 °S; Long: 60.25 °W	5	01/01/2012-10/21/2014	Local scale	Hourly
Venado Tuerto (VT)	Lat: 33.67 °S; Long: 61.97 °W	5 and 50	06/20/2012-05/16/2017	Local scale	Hourly
Anguil (ANG)	Lat: 36.50 °S; Long: 63.98 °W	5 and 50	03/15/2012-05/16/2017	Local scale	Hourly
Paraná (PAR)	Lat: 31.78 °S; Long: 60.48 °W	5 and 50	06/02/2015-05/16/2017	Local scale	Hourly
Monte Buey (MB)	Lat: 32.97 °S; Long: 62.45 °W	5	06/13/2012-12/31/2017	Local scale	Hourly
Ataliva (ATA)	Lat: 30.97 °S; Long: 61.33 °W	5	11/20/2012-10/21/2014	Local scale	Hourly

With the exception of the location of Monte Buey (MB, see location in Fig. 1), the rest of the sites have only 1 (one) sensor measuring. It is worth mentioning that the MB site located in the province of Córdoba in central Argentina is the core site for validation of satellite missions, like the Soil Moisture Active Passive (SMAP) over South America (Colliander et al., 2017). The core site of MB started measuring in 2012 and has 43 sensors that were used during an intensive field campaign with the idea of being comparable across different spatial scales. The sensor distribution is shown in Thibeault et al. (2015). However, as the number of sensors changed over time, a specific criterion was applied in order to get a spatial soil moisture value representative for that area. The criteria for selecting the most representative sensor in the MB site (i.e. being approximately representative of a 80 × 30 km^2^ area), focused first on finding the sensors with the longest time record and lowest mean relative difference (MRD, eq. 2).

(2) $${MRD}_{j}=\frac{\sum_{i}S_{j}-\bar{S}}{\bar{S}}$$

where S corresponds to the sensor number j, $\bar{S}$ corresponds to the spatial average among all sensors available for that day, and i index accounts for the number of measurements of sensor j used to perform the summation. The sensor identification number used, based on this criteria is the n°55. Following this analysis, it is worth noting that this location is the only site were the soil moisture values are representative of an area comparable to the LSMs/satellite spatial grid scale.

### 2.3 Land surface models

The Land Surface Models (LSMs) tested in this study are widely used by the scientific community and by operational national centers: GLDAS-Noah (GLDAS version 2.1, Rodell et al., 2004; Ek et al., 2003), ERA Interim-TESSEL (van den Hurk et al., 2000v), GDO-LISFLOOD (Roo et al., 2000) and the BHOA bucket model (Fernández Long et al., 2012). The GLDAS-Noah and ERA Interim TESSEL are reanalysis type products disseminated with a delay of 2–3 months, whereas GDO-LISFLOOD and BHOA are operational products available in near-real time.

The BHOA is a single layer hydrological bucket model that is locally driven by observations (e.g. precipitation) from conventional meteorological stations from the Argentinean Meteorological Service (SMN). All measurement sites described in Table 1 used in BHOA coincide with SMN meteorological stations, except for MB and ATA that use observations from Marcos Juarez (39 km) and Sunchales (13 km), respectively. It also incorporates local information about the soil types (e.g. field capacity and wilting point). Despite that BHOA has only one layer, a second simulation based on the 25 % of the field capacity (i.e. maximum water retained in the soil) was performed, in order to represent the surface soil moisture.

The GLDAS 2.1 Noah uses a combination of Global Precipitation Climatology Project (GPCP) and NOAA GDAS (National Oceanic and Atmospheric Administration, Global Data Assimilation System) atmospheric analysis and the U.S. Air Force Weather Agency’s AGRicultural METeorological modelling system (AGRMET) radiation fields. The Noah LSM is a 1-D column model, which explicitly simulates soil moisture, soil temperature, snow depth, etc. It is used since 1996 in the operational NCEP (National Centers for Environmental Prediction) models. The root depth corresponds to 0−100 cm depth of the LSM, except for the forest (0−200 cm) and tundra (0−40 cm) vegetation classes. The soil textures are based on the FAO (Food and Agricultural Organization) 16 categories.

The forcing variables used in the TESSEL LSM come from the ERA-Interim reanalysis variables to drive the model. This LSM is also a 1-D column model, where the rooting depth depends on the vegetation class, which is represented as a distribution percentage of roots. The highest percentage of roots is located within the 0−100 cm layer. For example, the forest vegetation class has the highest percentage of roots in the 100−289 cm layer (19 %) and the rest (81 %) in the 0−100 cm layer. The TESSEL LSM uses only one type of soil texture for the entire world, which is an important difference compared with the other LSMs.

The LISFLOOD is a GIS-based distributed hydrological rainfall-runoff-routing model designed to reproduce the soil moisture dynamic in three sub-layers (surface, root zone and sub-soil, Burek et al., 2013). The version used in this study has been operationally implemented for drought monitoring in GDO, and uses daily meteorological forcing maps derived from the ECMWF dataset as spatially resampled and harmonized by the JRC Monitoring Agricultural ResourceS (MARS) group.

The GLDAS 2.1 Noah and BHOA were temporal averaged from 12UTC to 12UTC. The ERA Interim TESSEL outputs correspond to 12UTC and LISFLOOD 00UTC. The spatial resolution and soil depth for each LSM are detailed in Table 1. The surface soil moisture depths from the different LSMs are: GLDAS-Noah 0−10 cm, ERA-Interim 0−7 cm, BHOA 0−15 cm. The depths for the deeper soil moisture, considered in this study as root zone soil moisture, are: GLDAS-Noah 0−100 cm, ERA-Interim 0−100 cm, BHOA 0−150 cm, LISFLOOD varying from 0−16 cm (Ataliva, ATA), Fig. 1), 0−75 cm (Venado Tuerto, VT) to 0−95 cm (Anguil, ANG).

### 2.4 Satellite estimations

The ESA-SM daily surface soil moisture product (version 4.2 accessed on May 2018, Liu et al., 2011, 2012; Dorigo et al., 2017) is here tested. In this study only, the passive product estimations are analysed, based on the results of Grings et al. (2015) over the Pampas region. The authors highlight the sensitivity to vegetation of the active estimations compared to the passive ones. Furthermore, the potential limitations of this product are documented in Dorigo et al. (2017) and Dorigo et al. (2012). In the period of interest (see Table 1), the passive product uses SMOS (2009 November-onwards), SMAP (2015 February-onwards), and AMSR2 (2012 July-onwards). The satellite soil moisture estimation depth varies from 0 to a few centimetres (Seneviratne et al., 2010).

## 3 Results

### 3.1 Surface soil moisture

The correlation coefficients for each of the 8 sites are reported in Table 3 for the surface datasets, along the average result for each model. These results show that the best performance is obtained for BHOA (0.74) followed by ERA-Interim (0.65), ESA-SM satellite estimations (0.64) and GLDAS-Noah (0.58). The average absolute bias is reported in Table 3 (last row) in order to prevent that positive bias in some locations compensates negative bias in other locations. The average absolute bias shows the lowest values for the ESA-SM estimation (13.0 %) and the highest for ERA-Interim (53.1 %). The RMSE average shows that BHOA, ESA-SM and GLDAS-Noah have similar values ranging between 32.8 % and 35.8 %, meanwhile the highest RMSE is observed for ERA-Interim (61.7 %). The mean non-systematic errors (unbRMSE) show similar percentages ranging from 24.9 % for GLDAS-Noah to 29.3 % for the ESA-SM, suggesting similar performance for all the models in term of non-systematic errors. The BHOA shows the highest correlation values over Pergamino (PER) with r = 0.89 and GLDAS-Noah the lowest correlation over Venado Tuerto (VT, r = 0.30). In regard to the BIAS, the ESA-SM shows the lowest absolute value in VT (0.78 %) and ERA-Interim shows the highest underestimation over Parana (PAR, −113.1 %). The ERA-Interim also shows the highest RMSE value in PAR (113.1 %), related to the high values of BIAS, and the lowest RMSE is shown by GLDAS-Noah in PER (16.5 %). The unbRMSE shows the highest value for ESA-SM (46.63 %) in ATA, and the lowest for BHOA in PER (12.37 %). Since some differences in performance could be due to the incompleteness of the time series, as the observational lengths of the records are different, we also contrasted the results only for the sites with records longer than 2 years (i.e. ANG, VT and MB). Also, in this case, the LSMs and ESA-SM still show a good performance, even if the BHOA shows still the highest correlation (0.76), followed in this case first by ESA-SM (0.63) and second by ERA-Interim (0.61), and last as above by GLDAS-Noah (0.47). Also, the BIAS and unbRMSE metrics averaged for ANG, MB and VT for the LSMs and ESA-SM stands as described above considering all sites. For the RMSE, the order also stands, but with an increment from 23.1 % to 35.9 % for GLDAS-Noah.

**Table 3:** Performance metrics for surface soil moisture. Spearman Correlation coefficient (“Corr”). The BIAS, RMSE, unbRMSE, are expressed in % relative to the individual mean values. All correlations are significant (p-value < 0.05).

Site Name	N	BHOA				GLDAS-Noah				ERA-Interim				ESA-SM			
		Corr	BIAS (%)	RMSE (%)	unbRMSE (%)	Corr	BIAS (%)	RMSE (%)	unbRMSE (%)	Corr	BIAS (%)	RMSE (%)	unbRMSE (%)	Corr	BIAS (%)	RMSE (%)	unbRMSE (%)
TAN	466	0.82	−37.8	44.9	24.3	0.63	−5.2	20.1	19.4	0.62	−56.0	63.2	29.3	0.74	−16.7	43.5	40.2
PER	450	0.89	18.7	22.4	12.4	0.76	4.7	16.5	15.8	0.71	−50.1	56.4	25.9	0.73	9.6	24.8	22.8
BAR	634	0.74	−15.8	33.6	29.7	0.65	−16.9	28.8	23.3	0.71	−43.5	51.3	27.2	0.67	13.9	21.6	16.6
VT	1626	0.76	−6.3	28.2	27.5	0.3	−22.0	48.6	43.3	0.66	−27.9	45.3	35.7	0.63	0.8	30.3	30.3
ANG	778	0.69	35.4	54.8	41.8	0.58	45.7	51.2	23.2	0.58	53.6	56.9	19.3	0.57	47.9	58.7	34.0
PAR	651	0.47	−9.4	26.4	24.7	0.51	−41.5	45.0	17.5	0.69	−113.1	114.2	15.7	0.64	−1.6	19.5	19.4
MB	1721	0.81	14.0	24.9	20.5	0.52	−40.0	53.0	34.8	0.6	−46.7	57.9	34.2	0.69	−2.2	24.7	24.6
ATA	680	0.73	8.6	27.2	25.8	0.7	9.0	23.6	21.8	0.67	−34.2	48.6	34.6	0.49	−11.4	48.0	46.6
Average		0.74	18.3	32.8	25.8	0.58	23.1	35.9	24.9	0.65	53.1	61.7	27.7	0.64	13.0	33.9	29.3

**Fig. 2:**
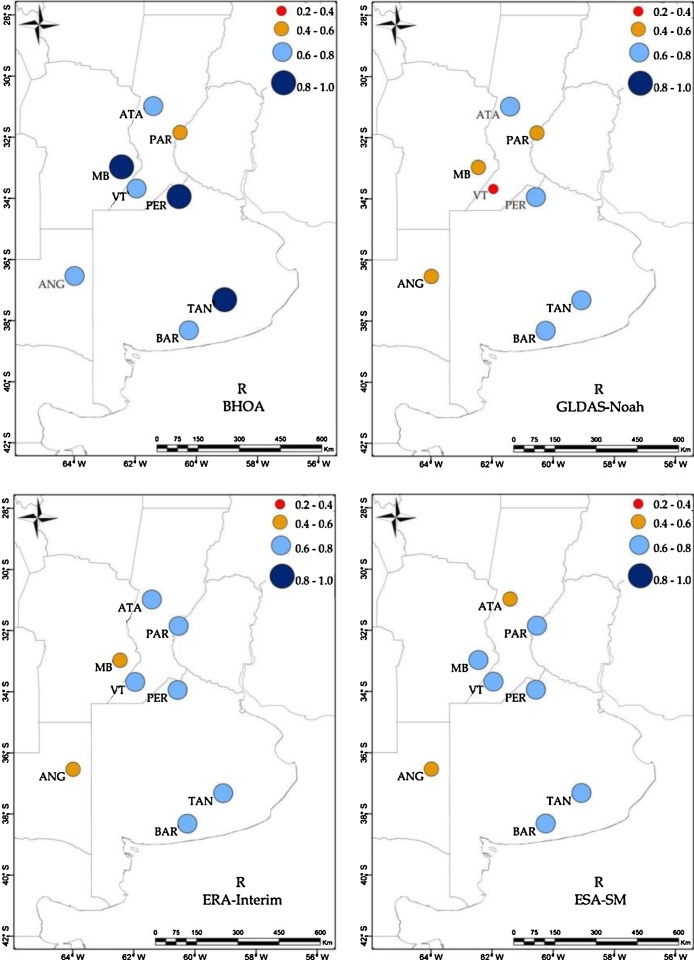
Spearman correlation computed between observed surface soil moisture and BHOA, GLDAS-Noah, ERA-Interim and ESA-SM.

**Fig. 3:**
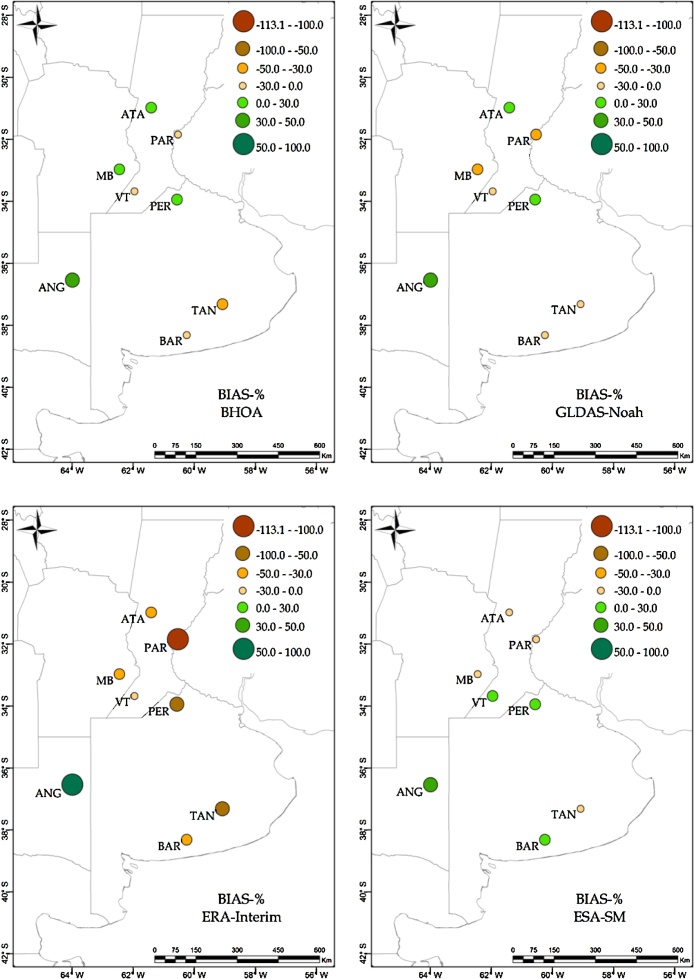
Surface soil moisture relative BIAS (%) for BHOA, GLDAS-Noah, ERA-Interim and ESA-SM.

Complementing Table 3, the spatial distribution of the correlations and relative BIAS are shown in Fig. 2, Fig. 3, respectively. In Fig. 2, a good agreement (r>0.6) is observed for the LSMs and ESA-SM over the Buenos Aires province (i.e. TAN, BAR and PER sites). For the rest of the locations, all soil moisture products show at least one site with correlation lower than 0.6. The lowest agreement is observed over ANG for GLDAS-Noah, ERA-Interim and ESA-SM, where only BHOA estimations tend to represent local conditions satisfactorily.

Unlike the rest of the LSMs and the ESA-SM estimations, in Fig. 3, ERA-Interim shows in general a dry BIAS (i.e. negative values) except for Anguil (ANG). GLDAS-Noah and ERA-Interim behave similarly in terms of errors, showing the driest BIAS in PAR and the wettest in ANG. Meanwhile BHOA and ESA-SM show the driest BIAS in TAN (see also Table 3). In particular, all products show a wet BIAS for the surface soil moisture in the ANG site and a dry BIAS over the PAR site.

**Fig. 4:**
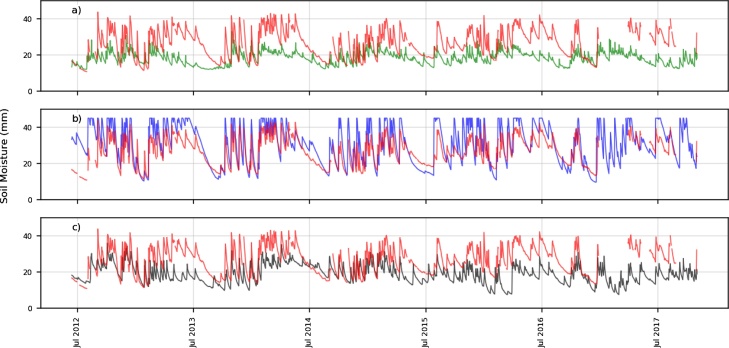
Daily in-situ measurements (red) and simulated surface soil moisture (mm) for Monte Buey (MB) a) ERA Interim, b) BHOA and c) GLDAS-Noah). Time range: June 2012-May 2017.

The two longest time series of surface soil moisture are shown in Figs. 4 (MB) and 5 (VT). The BHOA shows a similar, but higher, dynamical range compared to the in-situ measurements in MB (Fig. 4, panel b), whereas the GLDAS-Noah and ERA-Interim show a lower dynamic range. In addition, both LSMs show some peaks in soil moisture, likely related to precipitation events that were not observed locally (e.g. July 2015 in ERA-Interim). These discrepancies can be explained by the difficulties for global and regional forcing to correctly capture some local scale meteorological conditions.

**Fig. 5:**
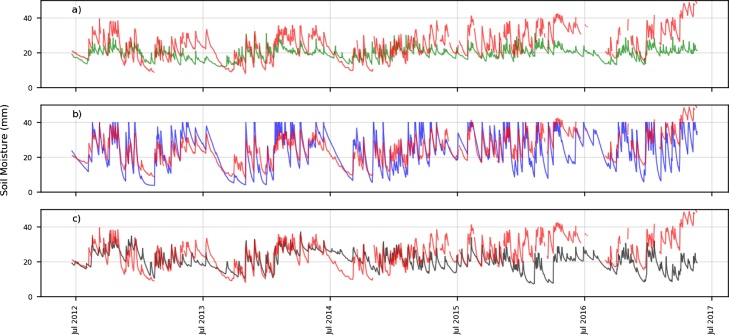
Daily in-situ measurements (red) and simulated surface soil moisture (mm) for Venado Tuerto (VT), a) ERA Interim, b) BHOA and c) GLDAS-Noah. Time range: June 2012-May 2017.

BHOA often reaches the saturation (with values of 45 mm for MB and 40 mm for VT), which is not observed in the other LSMs. Above these values the soil moisture surplus is considered as an excess and not retained in the soil (i.e. surface runoff). The so called dry-down periods (i.e. rate at which soil moisture dries out after a precipitation event) are better represented by BHOA compared to GLDAS-Noah and ERA-Interim in these locations. This effect is more evident for several days after July 2013 and prior to July 2014.

**Fig. 6:**
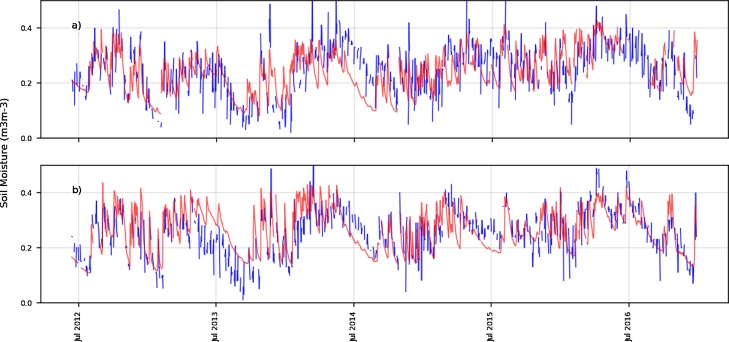
Satellite soil moisture (m^3^/m^3^) estimations (blue) and in-situ measurements (red) for a) Venado Tuerto (VT) and b) Monte Buey (MB). Time range: 2012-2016.

The ESA-SM (2012−2016) is able to adequately represent wet and dry events during this period of time (Fig. 6). Nevertheless, it shows a higher dynamical range over both VT and MB locations, similarly to the BHOA dynamics. It also shows a higher day-to-day variability, compared to the in-situ measurements and to the LSM simulations, which is expected given the thin layer observed by passive satellite products (i.e. from millimetres to few centimetres depth).

### 3.2 Root zone soil moisture

Analogously to the results reported for the surface layer, the results for root zone are summarized in Table 4. Overall, a decrease in the performance of the LSMs for the deeper layer is observed compared to the surface layer, although it is worth pointing out that at this soil depth the number of analysed sites is smaller compared to the surface layer (see Table 3). Comparing the two depths (surface vs. root zone) the averaged correlation values show lower values for the root zone: BHOA (0.69), GLDAS-Noah (0.07) and ERA-Interim (0.59). However, it is interesting to note that the BHOA shows higher correlation for VT and PAR, and lower for ANG compared to the surface layer. Thus, BHOA improves the correlation of the deeper soil moisture variability over VT and PAR compared to the surface soil moisture. The opposite behaviour is observed for GLDAS-Noah, which shows a considerable deterioration of the soil moisture representation compared to the surface layer. As for the surface layer, the ERA-Interim also shows a good agreement for the deeper layer. The LISFLOOD shows on average an intermediate agreement (0.38), being considerable better to GLDAS-Noah but inferior to ERA-Interim and BHOA.

**Table 4:** Performance metrics for root zone soil moisture. Spearman Correlation coefficient (“Corr”). The RMSE, BIAS, unbRMSE, are expressed in % relative to the individual mean values. All correlations are significant (p-value < 0.05) except for GLDAS-Noah.

Site Name	N	BHOA				GLDAS-Noah				ERA-Interim				LISFLOOD			
		Corr	BIAS (%)	RMSE (%)	unbRME (%)	Corr	BIAS (%)	RMSE (%)	unbRME (%)	Corr	BIAS (%)	RMSE (%)	unbRME (%)	Corr	BIAS (%)	RMSE (%)	unbRMSE (%)
VT	1618	0.81	−30.7	34.0	14.7	0.12	−45.1	58.7	37.5	0.63	5.2	16.0	15.1	0.48	21.3	25.7	14.3
ANG	779	0.55	31.3	40.1	25.1	0.13	34.7	47.1	31.9	0.58	70.8	71.8	12.1	0.29	68.7	70.5	15.8
PAR	644	0.71	−67.6	68.2	9.3	0.02	−70.1	73.2	21.2	0.57	−48.1	49.1	9.8	0.37	−16.6	21.4	13.4
Average		0.69	43.2	47.4	16.4	0.07	50.0	59.7	30.2	0.59	9.0	45.7	12.3	0.38	35.6	39.2	14.5

The relative BIAS (%) shows a deterioration of BHOA and GLDAS-Noah performance, almost doubling the BIAS values (in absolute terms) for the root zone compared to the surface soil moisture. The ERA-Interim shows an improvement of near 12 % in the performance of the root zone soil moisture. All LSMs show a wet (i.e. positive) BIAS for the root zone soil moisture over ANG and a dry BIAS over PAR, a behaviour also observed for the surface layer soil moisture. Over VT, ERA-Interim and LISFLOOD show a wet BIAS and GLDAS-Noah and BHOA show a dry BIAS. In particular, GLDAS-Noah and BHOA show a decrease in the BIAS for the root zone layer over ANG, and ERA-Interim shows a considerable increase of almost 20 % compared to the surface layer. The RMSE root zone soil moisture shows higher values for GLDAS-Noah and BHOA compared to surface layer, and the opposite is shown by ERA-Interim. The unbRMSE shows lower values for BHOA and ERA-Interim, and higher for GLDAS-Noah, for the root zone soil moisture compared to the surface layer.

**Fig. 7:**
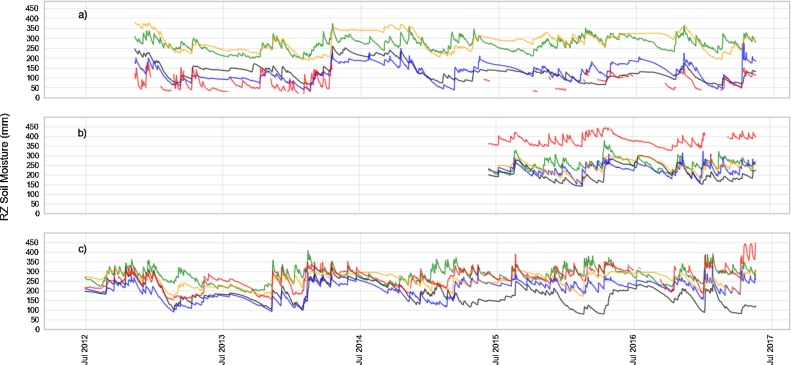
Root zone soil moisture (mm) for a) Anguil (ANG), b) Paraná (PAR) and c) Venado Tuerto (VT). BHOA (Blue), GLDAS-Noah (black), ERA-Interim (green), LISFLOOD (yellow) and in-situ measurements (red).

The temporal series of root zone soil moisture for ANG, PAR and VT are depicted in Fig. 7. Over ANG some soil moistures peaks (likely following precipitation events) are not captured by the LSMs (e.g. before July 2013 and during 2013–2014 austral summer). However, in general terms, the LSMs are able to reproduce the observed dry and wet periods. It is worth noting that around July 2013 the GLDAS-Noah, BHOA and LISFLOOD are not able to adequately represent the drying period. Consistently with the error metrics, LISFLOOD and ERA-Interim show a considerable difference in the mean value. In particular, LISFLOOD shows lower short term (i.e. daily) variability compared to the other LSMs and shows at least three periods with near constant high soil moisture values. This may be due to the real-time nature of the LISFLOOD model, and the absence of any corrections for the meteorological forcing in those periods. In the PAR site, all LSMs underestimated the mean soil moisture value. Also, a dry period that lasted for several months centred in July 2016, is not well represented by the LSMs. In general, in the VT site, a good performance of all LSMs is observed, in agreement with the performance metrics of Table 4. However, in particular the GLDAS-Noah simulations present a good agreement before 2015, but with a considerable reduction in the performance after this year.

### 3.3 Soil moisture anomalies and dry- wet event detection

In order to analyse the soil moisture anomalies (i.e. subtracting the seasonality of the time series, as in Eq. 1), the correlation between the observed anomalies, the LSMs and ESA-SM surface and root zone anomalies were calculated and summarized in Table 5. Average correlations of surface soil moisture anomalies (shown in Table 5) are always equal or weaker if compared with the soil moisture correlations (Table 3). This is observed for BHOA (0.74 vs. 0.69), GLDAS-Noah (0.58 vs. 0.58), ERA-Interim (0.65 vs. 0.59) and ESA-SM (0.64 vs. 0.53). But, over particular sites the correlation show equal or higher values of the surface soil moisture anomalies compared to the soil moisture series for: BHOA over VT and ANG; GLDAS-Noah over TAN, VT, PAR and MB; ERA-Interim over MB; and for ESA-SM over BAR.

**Table 5:** Spearman correlation coefficient for surface and root zone soil moisture anomalies. All correlations are significant with p < 0.05.

Correlation									
BHOA		GLDAS-Noah		ERA-Interim		LISFLOOD		ESA-SM	
Surface	RZ	Surface	RZ	Surface	RZ	Surface	RZ	Surface	RZ
0.74	–	0.63	–	0.61	–	–	–	0.59	–
0.75	–	0.53	–	0.68	–	–	–	0.57	–
0.67	–	0.59	–	0.69	–	–	–	0.68	–
0.79	0.80	0.64	0.58	0.62	0.54	–	0.50	0.51	–
0.69	0.63	0.54	0.36	0.56	0.48	–	0.44	0.56	–
0.44	0.69	0.55	0.50	0.51	0.49	–	0.50	0.39	–
0.73	–	0.58	–	0.62	–	–	–	0.58	–
0.71	–	0.55	–	0.44	–	–	–	0.32	–
0.69	0.71	0.58	0.48	0.59	0.50		0.48	0.53	

For the root zone layer the soil moisture anomalies show on average, except for ERA-Interim, higher correlation values compared to the soil moisture correlations: BHOA (0.69 vs. 0.71), GLDAS-Noah (0.26 vs. 0.48), ERA-Interim (0.59 vs. 0.50) and LISFLOOD (0.40 vs. 0.48). The improvement of the GLDAS-Noah correlation is remarkable. This improvement could be related with a poor representation of the soil moisture seasonality in particular years. In other words, GLDAS-Noah shows a better agreement in representing the short-term variability than the variability with the seasonal effect included. In general, the soil moisture anomalies show higher correlation values for the surface layer compared to the root zone layer, except for BHOA over the PAR and VT sites. A similar behaviour was observed over PAR and VT when comparing surface soil moisture against root zone soil moisture for BHOA in Section 3.2.

**Fig. 8:**
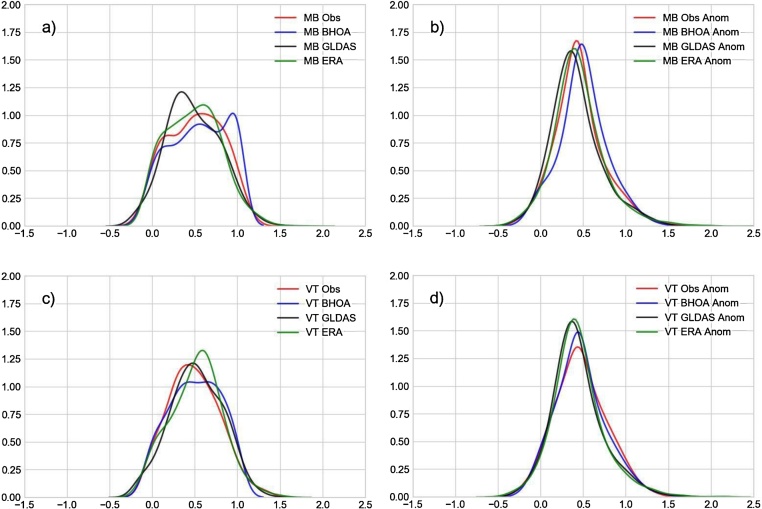
Kernel density function of surface moisture temporal series: a) MB and c) VT; and the soil moisture anomalies: b) MB and d) VT. Values were standardized using the 5th and 95th percentiles.

**Fig. 9:**
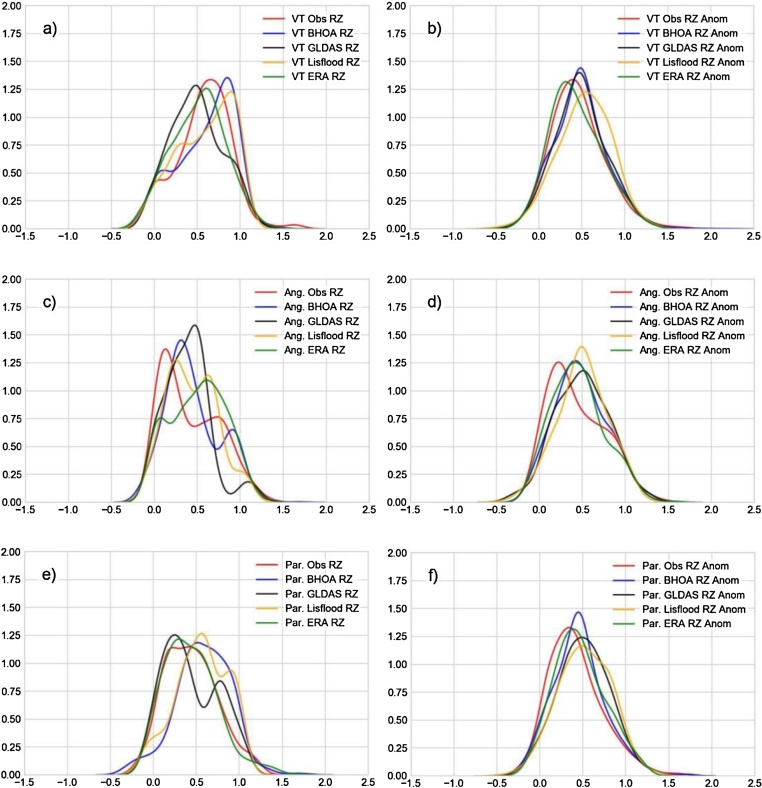
Kernel density function of the root zone soil moisture temporal series: a) VT, c) ANG e) PAR; and for the soil moisture anomalies: b) VT, d) ANG f) PAR. Values were standardized using the 5th and 95th percentiles.

The plots in Fig. 8, Fig. 9 show the density functions of the standardized surface soil moisture and the anomalies for the locations of MB and VT, and of the root zone soil moisture and the root zone anomalies for the locations of ANG, PAR and VT, respectively. As shown by Mo (2008) over different regions of the United States, soil moisture distributions can differ from a Gaussian/normal shape. Only in the VT site the soil moisture in-situ measurements (Fig. 8c) exhibit a type of Gaussian distribution; meanwhile the other locations show a bimodal shape. It is interesting to note that, there is a change in the distribution shape from the surface (normal-like) to the deeper layer (bimodal). The soil moisture anomalies show in general a normal distribution shape except for the root zone soil moisture in ANG (Fig. 9d). Analysing the LSMs, in some cases they show a different type of distribution compared to the in-situ measurements, as in case of GLDAS-Noah surface soil moisture in MB (Fig. 8a), GLDAS-Noah and LISFLOOD root zone soil moisture in PAR (Fig. 9e), and GLDAS-Noah root zone soil moisture in VT (Fig. 9a). In ANG all LSMs show for the root zone soil moisture a similar bimodal shape (Fig. 9c), except of ERA-Interim which presents a bimodal distribution but positively skewed with a longer right tail. The ESA-SM shows a similar distribution shape for the VT surface soil moisture, but for MB a more symmetric distribution shape instead of an observed bimodal distribution (not shown). In spite of the fact that soil moisture anomalies show a better agreement between the observed and simulated distributions, the Kolmogorov-Smirnov test for 2 samples was applied in order to quantify the differences. The null hypothesis of this test (i.e. that both samples are drawn from the same distribution) was rejected in most cases with p < 0.01, meaning that the LSM and the ESA-SM are not able to accurately reproduce the observed soil moisture and soil moisture anomalies distributions. The null hypothesis was not rejected for the surface soil moisture anomalies of BHOA over VT, and for ERA-Interim the soil moisture anomalies over MB (surface) and VT (root zone), and over PAR for the root zone soil moisture.

**Fig. 10:**
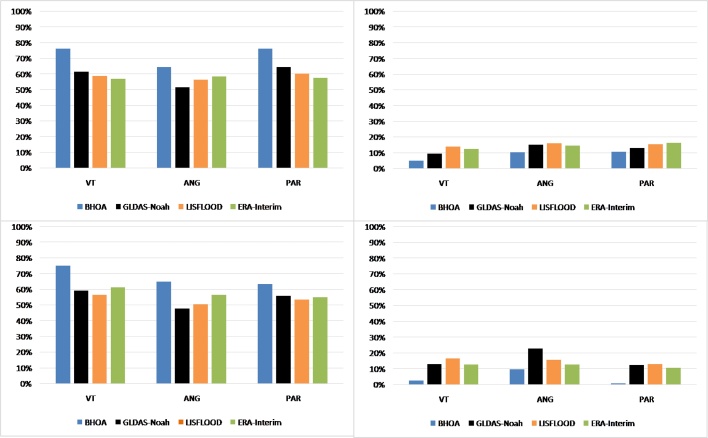
Hit rate for dry events (upper left panel) and for wet events (lower left panel), and False alarm (see details in Section 2.1) of the dry events (upper right panel) and wet events (lower right panel).

The LSMs ability in representing wet and dry events by means of the root zone soil moisture anomalies is shown in Fig. 10. Overall, the LSMs show similar values for detecting dry and wet events, with slightly higher performance for detecting dry events, except for ERA-Interim in VT. The LSMs show for the dry events lower errors over VT compared to ANG and PAR. For both dry and wet events, BHOA shows the best performance in detecting extreme values, with hit rate values above 63 % in all sites, showing the highest values over PAR and VT with a hit rate of 76 % for dry events. Comparing the detection of dry and wet events, in PAR the percentage of dry events is higher in contrast to the wet events for all LSMs. This behaviour is also observed in the location of VT for BHOA, GLDAS-Noah and LISFLOOD, but the opposite behaviour is shown by ERA-Interim. For the ANG site, all LSMs show equal or higher hit rate percentage of dry events compared to wet events. For the dry events, the errors averaged over all sites show similar values between all LSMs. In particular, BHOA shows the lowest false alarm value (9%) and LISFLOOD the highest (15 %), closely followed by ERA-Interim with 14 %. The highest false alarm percentage for dry events is observed in ANG (16 %) and PAR (16 %) by LISFLOOD and ERA-Interim, respectively. Averaging all LSMs over all three sites the ability for detecting dry events is of 62 % and 58 % for wet events, and the errors are 13 % and 12 %, respectively.

## 4 Discussion

The BHOA shows the overall best performance/agreement (for both surface and root zone), which can be attributed to the fact that this simple bucket model (compared to the other LSMs) uses observed local precipitation, radiation and soil properties data. This is not the case for the other LSMs, where global forcing may not be able to always capture the actual dynamic of local meteorological data. As an example, while the overall correlation for GLDAS-Noah surface soil moisture over the VT site is equal to 0.30, if the periods June 2012-April 2015 and May 2015–December 2017 are analysed separately, the correlation values are r = 0.60 and r = 0.37, respectively. These different results can be explained by some precipitation events (and consequent soil moisture peaks) shown by the LSM time series that were not observed in the ground record. These results are in line with several studies (e.g., Kato et al., 2007) documenting that the key aspects of the LSMs performance rely on the quality of the forcing data used (e.g., precipitation), the soil properties (e.g., soil texture) and on the soil physics parameterizations of the different LSMs.

Previous studies documented that 5 cm and 50 cm soil moisture measurements can be considered to be representative of the 0−10 cm and 0−100 cm depth soil moisture (e.g. Albergel et al., 2012). But, it must be kept in mind that among the LSMs the surface layer (root zone) varies from 0−7 cm (0−45 cm) to 0−15 cm (0−150 cm), which also not exactly matches with the observed measurements. Therefore, the difference in soil depth between measurements, LSMs and ESA-SM, as well as the different spatial resolution, could be another potential source of discrepancy between the outcomes of the various datasets. Also, the different soil moisture temporal averages of the LSMs (e.g., BHOA 12-12 UTC and ERA-Interim 00-00 UTC) could be a further source of discrepancy, introducing systematic differences related to the representation of the precipitation diurnal cycle. This could be more evident for the fast-responding surface soil moisture, whereas it may be less relevant for the root zone soil moisture due to the relative slower dynamics compared to surface soil moisture.

Our study shows results that are consistent with the ones reported in Grings et al. (2015) in regard to the dynamic range of the surface soil moisture over the MB site. The authors observed that SMOS estimations show a greater dynamic range and GLDAS-Noah shows a systematic underestimation compared to the observed soil moisture for 2012−2014. However, they mention that GLDAS-Noah shows higher correlations than SMOS (used in the ESA-SM product) passive estimations. In this study, where a longer record was analysed, the ESA-SM satellite estimations show higher correlation than GLDAS-Noah over MB, a difference possible related to the forcing uncertainties in the model, as mentioned above. It is important to further stress that MB is the only site were the soil moisture values are representative of an area comparable to the LSMs/satellite spatial grid scale.

In general, it seems that the dynamic range of the observed surface soil moisture is overestimated by BHOA and ESA-SM and underestimated by GLDAS-Noah and ERA-Interim over both MB and VT sites. The most adequate representation is given by BHOA, which could be related to the use of more accurate soil hydraulic parameters (e.g. field capacity and wilting point). These parameters seem to be critical to the other LSMs, showing important differences in the soil moisture dynamic range, which led to a strong bias. In particular, in Albergel et al. (2012) the TESSEL LSM used in ERA Interim is compared against a new version of the LSM, the H-TESSEL. One of the main improvements of H-TESSEL is related to the utilization of a global texture data, because the TESSEL only uses a specific value of soil texture. An obvious consequence of using a constant value of soil texture is an inadequate representation of the soil moisture dynamic range. In this study, the ERA Interim (TESSEL) also had problems to adequately reproduce this feature, showing the highest BIAS values among the different LSMs. Besides this, ERA-Interim showed good results. Albergel et al. (2012) also showed that ERA-Interim tends to overestimate the soil moisture values over dry regions. In this study, all LSMs (except BHOA) showed an overestimation of the soil moisture values over ANG, which is the driest site considered with an annual precipitation of 632.2 mm/year. However, in our study there is not a clear impact of the soil moisture overestimation on the correlations as documented by Albergel et al. (2012) over the African Monsoon Multidisciplinary Analysis (AMMA) network in West Africa. In the AMMA network a large overestimation of ERA-Interim soil moisture led to a smaller variability, and consequently to poor correlation. In general, the BIAS errors can be corrected/calibrated (i.e. bias correction), but this type of correction has no effect when the overestimation reaches values near the field capacity or higher, as was the case of the AMMA network.

In the case of GLDAS-Noah, considerable error amplification from the surface soil moisture to the root zone soil moisture was observed. Based on this study, it is not possible to determine if the source of error is mainly related to the data forcing or also enhanced by the soil parameter uncertainties and/or physical parameterizations of the model. The other LSMs also show, on average, lower correlations for the root zone compared to the surface soil moisture. However, BHOA shows higher correlation over two (i.e. VT and PAR) of the three sites and ERA-Interim shows the same correlation value over one site (i.e. ANG). A similar behaviour is documented by Albergel et al. (2012) over particular soil moisture networks with lower correlation values for the root zone compared with surface soil moisture. But the authors observed that, on average, the root zone shows higher correlation values.

The LSMs and the ESA-SM have difficulties in reproducing the point scale soil moisture (i.e. surface and root zone) frequency distributions. Nevertheless, the shape of the distributions of soil moisture anomalies showed more resemblance with the in-situ measurements than the soil moisture series. Additionally, over particular sites some of the LSMs (i.e. BHOA and GLDAS-Noah) showed an improvement in the correlation values of the root zone soil moisture anomalies (i.e. short-term variability) compared to the soil moisture time series. The latter results, related to the root zone anomalies, were reflected in the good degree of confidence shown by all LSMs in the ability of detecting anomaly dry and wet events. In particular, the wet events showed higher errors, which can be presumably attributed to, for example, underestimation of local intense precipitation events or missing of some local events.

## 5 Conclusions

The LSMs and the ESA-SM satellite soil moisture estimations showed to be able to represent the main characteristics of the local soil moisture variability. In general, soil moisture products based on LSMs focus on anomalies in order to detect excess or deficit soil moisture conditions. But this is not the case with BHOA, which was designed to represent the water content available for crops. In this sense, it is encouraging for the different products, as BHOA is able to better represent the soil moisture dynamic range and variability compared to the other LSMs, and GLDAS-Noah, ERA-Interim and LISFLOOD are able to adequately represent the soil moisture anomalies over the Pampas region. In addition to the LSM results, also the ESA-SM anomalies proved to be valuable. Nevertheless, the reliability of the results obtained in this study could be sensitive to different factors, such as differences in soil depth between datasets, differences between point scale measurements and area-average estimates (i.e. LSM spatial grid), as well as differences in LSM and satellite spatial resolutions and temporal averages.

Accurate forcing data and soil parameters are critical to substantially improve the ability to detect dry and wet events. In fact, even with LSMs forced with local observations, the percentage of false alarms is not negligible (i.e. about 9% for dry events). Thus, suggesting to further investigate the conditions under which the LSMs are most likely to reproduce or miss the deficit conditions in order to recommend improvements on model parameterization that may still be needed even for local models. A further evaluation will focus on the impact of these differences, among the LSMs, in representing dry events in the framework of a Combined Drought Index specifically designed for this region.

## Declaration of Competing Interest

The authors report no declarations of interest.

## Footnotes

1 Mention of trade names does not constitute endorsement by the authors.
